# An Evidence-Based Treatment of Myofascial Pain and Myofascial Trigger Points in the Maxillofacial Area: A Narrative Review

**DOI:** 10.7759/cureus.49987

**Published:** 2023-12-05

**Authors:** Sattam S Alshammari, Salman Amin, Ammar Ahmed Siddiqui, Yasser Riaz Malik, Abdullah Faraj Alshammari, Junaid Amin

**Affiliations:** 1 Department of Preventive Dental Sciences, College of Dentistry, University of Ha’il, Ha’il, SAU; 2 Department of Oral and Maxillofacial Surgery, College of Medicine and Dentistry, The University of Lahore, Lahore, PAK; 3 Department of Preventive Dental Sciences, College of Dentistry, University of Ha'il, Ha'il, SAU; 4 Department of Basic Dental and Medical Science, College of Dentistry, University of Ha’il, Ha’il, SAU; 5 Department of Physical Therapy, College of Applied Medical Sciences, University of Ha’il, Ha’il, SAU

**Keywords:** myofascial pain, evidence-based treatment, temporomandibular disorders, trigger points, physical therapy

## Abstract

Myofascial pain (MFP) is characterized by localized pain in the maxillofacial region attributed to the presence of hypersensitive spots known as trigger points (TrPs). This condition is particularly prevalent in the maxillofacial area, warranting a comprehensive examination of evidence-based management techniques. This review aims to equip healthcare professionals with a more profound insight into evidence-based MFP management techniques, facilitating improved patient care and treatment outcomes. In this review, we conducted a thorough literature search using Google Scholar, Scopus, Web of Science (WOS), and MEDLINE, with the keywords "Myofascial pain syndrome," "Pain," and "Orofacial pain." Articles were selected based on their relevance to the study's objective.

Pharmacological interventions, such as analgesics and muscle relaxants, are frequently prescribed. Additionally, a range of non-pharmacological modalities, including transcutaneous electrical nerve stimulation (TENS), ultrasound therapy, topical applications, dry needling, TrP injections, oral myofunctional therapy, and stretching exercises, have demonstrated efficacy in MFP management. The authors hope to give clinicians a more thorough understanding of the therapies for MFP by conducting a rigorous evidence-based evaluation of pharmacologic and non-pharmacological treatments. Our findings support the use of a combined approach that integrates both pharmacological and non-pharmacological strategies for the holistic management of TrPs.

## Introduction and background

Myofascial pain (MFP) is a complex condition characterized by localized muscle pain attributed to the presence of tender trigger points (TrPs) within the musculature [1]. These trigger points, or TrPs, represent a focal area of hypersensitivity within a muscle, and they play a pivotal role in the manifestation of MFP. While the clinical diagnosis of MFP primarily relies on the recognition of motor and sensory features, it is important to note that not all these symptoms need to be concurrently present for a diagnosis [2,3]. Furthermore, TrPs may exhibit autonomic characteristics, but these features are not imperative for clinical identification. Instead, the presence of taut bands within a muscle stands as a fundamental hallmark distinguishing a TrP from general muscle tenderness [4].

The development of TrPs is understood through two key concepts. Firstly, some factors directly traumatize a muscle, either through direct injury or recurrent micro traumas stemming from habits that generate tension within the muscle [5]. Common examples encompass poor postural habits and parafunctional oral habits, with poor posture representing a frequent contributor. Secondly, some factors weaken and predispose a muscle to the formation of TrPs. Such factors may include nutritional imbalances, structural disharmony, insufficient exercise, sleep disturbances, or underlying joint disorders. The intricate clinical features of MFP can make diagnosis challenging [2]. To suspect an MFP diagnosis, healthcare practitioners must adeptly discern pain descriptors, associated symptoms, and potential modifying factors. Upon suspecting MFP, practitioners rely on their knowledge and palpation skills to locate and assess possible TrPs. The gold standard for detecting taut bands in muscles with TrPs is palpation, which requires practitioners to undergo training and develop precise skills for accurate identification of these tense areas [6].

Notably, the prevalence of TrPs is high within the maxillofacial region, and symptoms of MFP in this area are predominantly associated with muscles such as the masseter, temporalis, and medial pterygoid. Importantly, the existence of MFP transcends age, with peak incidence observed between 40 and 50 years of age, and it displays a higher occurrence among women [7].

In this article, we take a deep dive into the multifaceted realm of MFP, with a keen focus on exploring the nuanced strategies for treating TrPs in the maxillofacial region. By thoroughly reviewing the most recent and relevant literature, our primary objective is to equip clinicians with a thorough, contemporary, and evidence-based understanding, thereby empowering them to effectively manage MFP in their practice. The research question was: "What are the evidence-based management techniques for MFP?

## Review

Methods

Search Strategy

In this review, we conducted a thorough literature search using Google Scholar, Scopus, Web of Science (WOS), and MEDLINE, with the keywords "myofascial pain syndrome," "pain," “trigger points," "tender trigger points,” and "orofacial pain." Articles were selected based on their relevance to the study's objective. The following search strategy was used: ("Myofascial Pain Syndrome") AND ("Pain" OR "Discomfort" OR "Ache" OR "Soreness") AND ("Trigger Points" OR "Tender Points") AND ("Orofacial Pain")

Inclusion/Exclusion Criteria

Studies that were published in English and used non-invasive treatment approaches including pharmacological and non-pharmacological intervention were included in the current review. Studies not published in the English language with insufficient information regarding treatment interventions or outcomes were excluded.

*Literature Search* 

**Figure 1:**
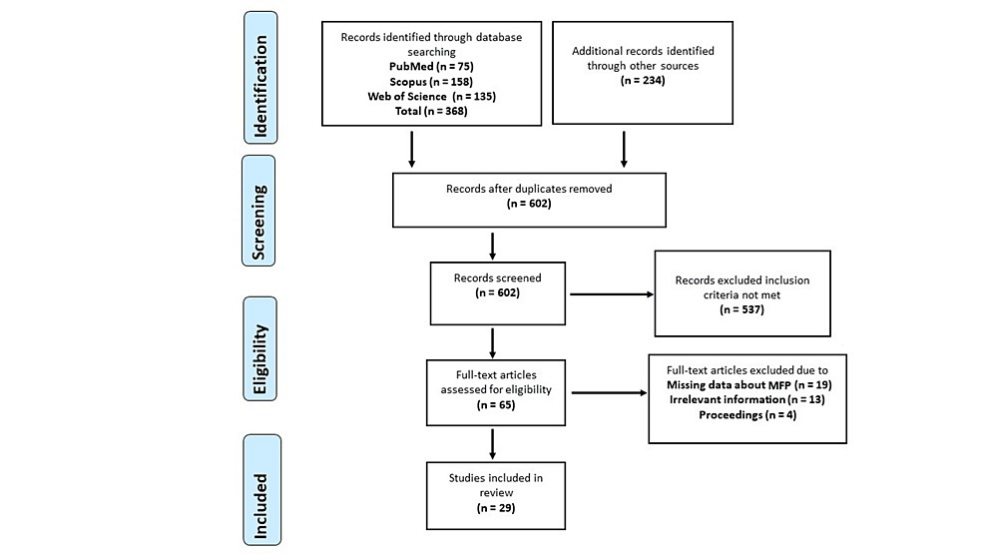
Flowchart showing the selection process of the studies WOS: Web of Science; MFP: myofascial pain

A comprehensive search yielded 602 studies from databases such as Scopus (158), Web of Science (135), and PubMed (75). Furthermore, 234 additional reports were discovered through supplementary sources, including Google Scholar and ResearchGate, and by examining the reference lists of the initially sourced articles. Following a rigorous screening process, 537 studies were excluded due to not meeting the inclusion criteria, including 19 studies with insufficient data about MFP, 13 studies that were considered irrelevant, and four studies that were conference proceedings. Ultimately, the review incorporated 29 studies (12 pharmacological interventions and 17 non-pharmacological interventions) that met the specified criteria (Figure 1).

Pharmacological interventions 

Analgesics

Non-steroidal anti-inflammatory drugs (NSAIDs) are highly effective in managing mild to severe inflammatory pain. They work by inhibiting the activity of cyclooxygenase (COX), which subsequently reduces the production of prostaglandins, thromboxane, and prostacyclin. This makes NSAIDs a valuable choice for treating various inflammatory conditions which are also proven for the management of acute musculoskeletal disorders [8].

Comparatively, opioid analgesics have shown similar effectiveness in addressing inflammatory pain when compared to NSAIDs [9]. Codeine is one of the most commonly prescribed opioid analgesics. However, opioids are not as frequently used as NSAIDs due to their propensity to cause a wide range of adverse effects and the risk of dependence and addiction associated with long-term use [10].

In cases where NSAIDs alone prove insufficient, or when prolonged usage is required for managing moderate to severe pain, a combination approach can be employed. This involves combining an opioid analgesic or a steroid with an NSAID. This combination therapy can provide enhanced pain relief while potentially mitigating some of the side effects associated with individual opioid use [8].

Muscle Relaxants

Tizanidine, a centrally acting alpha2 agonist, offers a valuable therapeutic option for alleviating spasticity. Its mode of action involves enhancing pre-synaptic inhibition of motor neurons, operating within both the brain and the spinal cord [11]. In a clinical treatment study, patients experiencing acute painful muscle spasms found relief with a regimen of 2 mg of tizanidine hydrochloride three times a day. This treatment resulted in a significant reduction in the frequency and intensity of painful muscle spasms [12]. The extensive research conducted by Hutchinson et al., involving 2251 participants from around the world, demonstrated the effectiveness of tizanidine hydrochloride in managing painful muscular spasms. A remarkable 89 percent of the patients in this global study reported positive outcomes, affirming the therapeutic value of tizanidine in the treatment of painful muscle spasms [12]. 

Antidepressants

Tricyclic antidepressants represent a class of medications known for their multifaceted therapeutic effects, including anti-inflammatory, anti-fibromyalgia, and anti-neuropathic pain properties [13]. Despite their wide use, there has been limited research focused specifically on their effectiveness in treating MFP. One study delved into the therapeutic potential of amitriptyline in managing chronic tension headaches, aiming to elucidate its mechanism of action. In this crossover research, amitriptyline demonstrated its therapeutic significance by yielding statistically significant results. It notably reduced pain (p = 0.01) and markedly alleviated headache severity when compared to a placebo [14]. Similarly, another investigation explored the use of amitriptyline in the context of chronic temporomandibular pain. After a six-week treatment period, all pain levels saw statistically significant reductions [15]. While there is currently no concrete evidence supporting the use of these therapies for MFP management, a growing body of research suggests their effectiveness in the realm of chronic pain syndromes, particularly when conventional treatments fall short [16].

Botulinum Toxin

Botulinum injections offer a multifaceted approach to relieving discomfort. They work by promoting increased blood flow to affected muscles, thus aiding in the release of nerve fibers that may have been compressed due to abnormal muscle contractions. This dual action contributes significantly to pain alleviation [17]. Moreover, botulinum injections exert immediate effects. This can be attributed to various mechanisms at play. Firstly, these injections lead to the direct release of endogenous endorphins, the body's natural pain-relieving substances. Additionally, they induce changes in the central neurotransmitter balance, primarily through the local inhibition of pain peptides originating from sensory ganglions and nerve terminals. Furthermore, botulinum injections exhibit anti-inflammatory properties, and they counteract the actions of glutamate, an excitatory neurotransmitter, which can further contribute to their pain relief effects [18].

Local Anesthetic

Lidocaine functions as a non-specific sodium channel blocker, which, in turn, stabilizes neuronal cell membranes, thereby hampering the initiation and transmission of nerve impulses. However, it is important to be aware of potential side effects associated with lidocaine injections, including anaphylaxis, central nervous system depression, epilepsy, and arrhythmia [19]. In a study conducted by Affaitati et al., the efficacy of a lidocaine injection was compared to that of a lidocaine patch in addressing MFP. Surprisingly, both methods proved equally successful in reducing pain [20]. However, it was noted that patients reported a lower level of treatment unpleasantness when utilizing the lidocaine patch. This finding sheds light on the overall patient experience and preferences regarding these pain management approaches [21].

Non-pharmacological interventions

Behavioral Therapy

Behavioral therapy serves as a valuable conservative approach in the management of temporomandibular disorders (TMDs). These disorders are characterized by a complex interplay of factors, where psychological elements and parafunctional hyperactivity play pivotal roles in their pathophysiology. It is increasingly evident that mood changes have a significant influence on pain threshold levels in individuals, contributing to the intricate connection between pain responses and psychological factors [22].

The relationship between psychological factors and pain responses in TMDs is multifaceted and often challenging to decipher. Experimental studies have underscored the importance of mood alterations in shaping an individual's experience of pain. However, the precise mechanisms and interactions involved in this complex relationship continue to be a subject of ongoing research.

Emerging evidence suggests that a comprehensive approach, addressing both psychological and dental factors, yields superior outcomes in the management of TMDs. This holistic strategy incorporates interventions such as intraoral appliances, biofeedback training, and stress reduction techniques. Remarkably, studies indicate that the combined management of these factors surpasses the results obtained by addressing either intraoral appliances or stress management in isolation [23].

Dry Needling

TrP dry needling is a therapeutic technique where an acupuncture-like needle is precisely inserted into the skin and muscle at the site of TrPs. These needles are removed once the TrPs have been successfully inactivated [24]. Typically, stretching exercises complement the dry-needling process.

This method often induces a localized twitch response, disrupting the activity of the motor end-plate, which, in turn, creates an analgesic (pain-relieving) effect. Additionally, the tight bands of actin-myosin linkages within the muscle tend to release due to the localized twitch response and the subsequent incorporation of stretching exercises [25]. The dry needling is equally effective compared to TrP injections, establishing its eligibility as a primary treatment for acute cases [2].

It is noteworthy that, in two separate trials, both lidocaine and botulinum toxin injections demonstrated more effective pain reduction when compared to dry needling. These findings underscore the potential advantages of alternative treatments in managing pain associated with TrPs [25].

Injection Therapy and Electro Manual Therapeutic Approaches

In the management of MFP, TrPs within taut muscle bands are typically addressed through a combination of injection therapy and physical interventions [19]. Traditional treatment modalities encompass a spectrum of approaches, which include manual therapies, electrical stimulation, heat and cold applications, local anesthetics, and needle injections.

Manual therapies often involve techniques such as stretching, massage, ischemic compression, and myofascial release. These methods are aimed at alleviating muscular tension and discomfort associated with TrPs. Among these approaches, "spray and stretch" is a commonly used technique for managing TrPs due to its effectiveness in providing immediate pain relief [19,21]. The spray and stretch approach requires the passive extension of contracted muscles with the simultaneous application of vapocoolant such as dichlorodifluoromethane-trichloromonofluoromethane or ethyl chloride [26]. Pain relief with therapeutic ultrasound is linked to the uptake of pain mediators by increased blood circulation, alteration in nerve conduction, or changes in cell membrane permeability that reduce inflammation [27].

Myofascial release, on the other hand, is a therapeutic method focused on repositioning surface tissues over underlying structures. The objective is to enhance mobility and mitigate the subcutaneous stiffness commonly seen in panniculosis. These diverse treatment options collectively offer a comprehensive strategy for addressing MFP by directly targeting the source of discomfort within the muscle tissue [28].

Transcutaneous Electric Nerve Stimulation (TENS)

TENS has been used successfully for the treatment of acute and chronic conditions of MFP. TENS relieves the pain by increasing the endogenous opiates in the circulation by its peripheral mechanism or central effects or autonomic response. It also increases blood circulation and oxygen supply eliminating toxic metabolic products. The TENS pain-relieving effect could be due to a peripheral mechanism or central actions that boost circulating endogenous opiates or regulate autonomic response [29]. Results were recorded by El Fatih et al. that the ultrasound group showed a higher success rate with pain improvement of 93.3 percent while the TENS showed only 53.3 percent success [30].

Oral Myofunctional Therapy

A variety of exercise regimens have been recommended for the treatment of TMDs. However, a specialized therapeutic approach aimed at restoring stomatognathic function, known as oral myofunctional therapy (OMT), has gained prominence. OMT primarily revolves around a series of carefully designed mouth exercises and interventions, offering a unique strategy for TMD management [21,31].

Notably, in a study conducted, individuals undergoing OMT experienced significant benefits. They reported a substantial reduction in pain sensitivity when the masticatory muscles were palpated. Additionally, measurements of mandibular range of motion displayed marked improvements. These findings underscore the potential efficacy of OMT in addressing TMD-related issues, suggesting that targeted exercises and therapies can contribute positively to the management of this condition [32].

The oral myofascial treatment encompasses the alleviation of pain and the relaxation of muscles in the jaw, shoulders, and neck, emphasizing the establishment of an appropriate resting posture for the mandible. The treatment involves promoting lubrication of the temporomandibular joint (TMJ) and ensuring controlled and symmetrical mobility of the mandible. Furthermore, it incorporates the application of both active and passive exercises aimed at enhancing mandibular mobility, including exercises for mouth opening and closing. Distinct exercises targeting mobility and isometric strength are applied individually for the tongue, lips, and cheeks. Isometric exercises for the tongue entail pressing the tip against a tongue depressor. Lip isometrics involve using tools like patakara or an oral rehabilitation device to train lip closure. Cheek isometrics consist of pressing palms into the cheeks while attempting to lift them upward [32].

Splint Therapy

The predominant approach to pain management for patients with TMD often involves the utilization of occlusal splints, either as a standalone treatment or in combination with other therapeutic interventions [33,34]. The choice of occlusal splints depends on the specific issue being addressed, as well as the individual needs of the patient. A dentist evaluates the condition and recommends the most appropriate type of splint for the situation. There are several types of occlusal splints: 1. Soft splints*:* Made from soft materials such as silicone or rubber and provide cushioning for the teeth and jaw, 2. Hard splints*:* Constructed from rigid materials like acrylic, 3. Dual laminate splints*:* Combines both soft and hard materials and provides comfort while maintaining durability, 4. Anterior Repositioning Splints (ARS)*: *Designed to reposition the lower jaw (mandible) forward and used to alleviate symptoms of TMDs and certain types of jaw misalignment, 5. Flat Plane Splints*: *Have a flat surface that allows the teeth to contact evenly, 6. Stabilization splints:Aimed at stabilizing the jaw and reducing muscle hyperactivity, 7.* *Neuromuscular splints: Focus on achieving a balanced relationship between the muscles and the TMJ and designed to relax the jaw muscles and optimize jaw function, and 8.* Michigan splints: *Full-coverage splints that cover all the teeth in one arch and are often used for diagnostic purposes [33].

The precise mechanism underlying the action of occlusal splints remains a subject of ongoing investigation and has not been fully elucidated [35,36]. Numerous theories have been proposed in an attempt to shed light on how occlusal splints exert their effects. These theories encompass various mechanisms, including *1. *Occlusal Condition Alteration*:* Some suggest that occlusal splints may work by modifying or enhancing the occlusal condition, potentially reducing the stress and strain on the TMJ and associated structures [37], 2. Peripheral Impulse Modification*:* Another theory postulates that occlusal splints could influence peripheral impulses, both motor and afferent, transmitted to the central nervous system, ultimately impacting pain perception [37], 3. Vertical dimension adjustment*:* Alterations in the vertical dimension, which can occur with occlusal splints, might contribute to changes in TMJ condylar position, potentially leading to symptom relief [37], 4. Enhanced cognitive awareness*: *It is also proposed that occlusal splints might improve cognitive awareness of jaw positioning and habits, leading to behavioral adjustments that mitigate TMD symptoms [38], *5. *Placebo effect:The psychological component of relief should not be underestimated. For some patients, the use of occlusal splints may trigger a placebo effect, where the expectation of improvement itself leads to symptom alleviation [38], and 6. Regression to the Mean*:* In some cases, TMD symptoms may naturally fluctuate, and patients may seek treatment during a period of heightened discomfort. Subsequent improvement may be attributed to the natural regression of symptoms [38,39].

In summary, the precise workings of occlusal splints in TMD management remain a topic of ongoing exploration. It is likely that multiple factors, both physical and psychological, contribute to the relief experienced by patients using occlusal splints. As research continues, a clearer understanding of their mechanisms will likely emerge.

Low-Level Laser Therapy

Low-level laser therapy (LLLT), also known as cold laser therapy or photobiomodulation therapy, involves the use of low-intensity lasers or light-emitting diodes (LEDs) to stimulate cellular function and promote tissue healing. The results of a systematic review indicate that LLLT appears to be efficacious in alleviating pain among individuals with temporomandibular MFP, supported by evidence of moderate quality [40].

Findings

The included studies reported a range of pharmacological interventions, such as analgesics and muscle relaxants, as well as non-pharmacological modalities, including TENS, ultrasound therapy, topical applications, dry needling, TrP injections, oral myofunctional therapy, LLLT, and stretching exercises, are available (Table 1). We conducted a quality assessment of the included articles using the JBI criteria, and two authors independently evaluated each study. Studies with a JBI score ranging from 20 to 49% were classified as having a high risk of bias, those with scores between 50-79% were considered to have a moderate risk of bias, and those scoring 80-100% were categorized as having a low risk of bias (Table 1).

**Table 1:** Characteristics and quality assessment of included studies ***low risk of bias ** moderate risk of bias

Study	Design	Intervention	Outcome	Quality assessment
Cigerim, and Kaplan (2023) [8].	Randomized, double-blind, controlled trial	Naproxen-codeine, naproxen, dexamethasone, and naproxen	Significant reduction in pain intensity in all three groups Both naproxen-codeine and naproxen-dexamethasone demonstrated efficacy in treating myofascial pain.	***
Jayadev et al. (2014) [9].	Observational study	Analgesics	High prevalence of antibiotic and non-narcotic analgesic prescription for pulpal and periapical pathologies	***
Şermet et al. (2012) [10].	Observational study	Non-steroidal anti-inflammatory drug (NSAID)	High prevalence of non-steroidal anti-inflammatory drug (NSAID) prescription for dental pain	**
Annaswamy et al. (2011) [11].	Review	Medications, topical agents, and modalities	Most interventions for myofascial pain lack adequate evidence, while certain medications, topical agents, and modalities show moderate to strong support.	N/A
Malanga et al. (2002) [12].	Randomized, double-blind, controlled trial	Tizanidine	Tizanidine showed significant reduction in pain intensity and improvement in function in myofascial pain.	***
Micó et al. (2006) [13].	Review	antidepressants	Pain and depression share common biochemical mechanisms, implying that antidepressants possess a legitimate analgesic effect.	N/A
Bendtsen and Jensen (2000) [14].	Randomized, controlled trial	Amitriptyline	Amitriptyline was effective in significant reduction in myofascial tenderness	**
Plesh et al. (2000) [15].	Case series	Amitriptyline	Amitriptyline Improved the pain and function in patients with temporomandibular disorders	**
Desai et al. (2013) [16].	Review	Physical therapy and self-care measures.	Myofascial pain can be effectively addressed through diverse treatments, encompassing medications, physical therapy, and self-care measures.	N/A
von Lindern et al. (2003) [17].	Case series	Botulinum toxin	Botulinum toxin showed improvement in pain and function in patients with chronic facial pain associated with masticatory hyperactivity.	**
Chaurand et al. (2017) [18].	Randomized, controlled trial	Botulinum toxin	Botulinum toxin demonstrated effectiveness in reducing pain and enhancing jaw function in individuals with myofascial pain.	***
Galasso et al. (2020) [21].	Review	Lidocaine	The efficacy of a lidocaine injection and that of a lidocaine patch equally successful in reducing pain.	N/A
Bogart et al. (2007) [22].	Randomized, controlled trial	Group cognitive behavior therapy	Significant reduction in pain intensity and improvement in function.	***
Michelotti et al. (2004) [23].	Randomized, controlled trial	Home physical therapy regimen	A home-based physical therapy routine proved more effective than sole patient education in alleviating pain and enhancing jaw function in individuals with myofascial jaw muscle pain.	**
Kietrys et al. (2013) [25].	Systematic review and meta-analysis	Dry needling	Dry needling is an effective treatment for upper-quarter myofascial pain.	***
Koole et al. (2020) [26].	Randomized, controlled trial	Spray and stretch technique	The spray and stretch technique resulted in a notable increase in maximal mouth opening, particularly in individuals with orofacial pain, and the effect was more pronounced in women than in men.	***
Xia et al. (2017) [27].	Systematic review and meta-analysis	Ultrasound therapy	Current evidence indicates a notable impact of ultrasound therapy on pain in patients with myofascial pain, although it does not appear to have a significant effect on range of motion.	***
Werenski (2011) [28].	Review	Myofascial release techniques	Myofascial release treatments, characterized by diverse applications, show notable effectiveness when addressing injuries related to myofascial tissue. The treatments vary in factors such as pressure, duration, motion, and tension, leading to varied results and outcomes.	N/A
Hou et al. (2002) [29].	Randomized, controlled trial	Various physical therapeutic modalities: hot pack, transcutaneous electric nerve stimulation (TENS), stretch with spray.	Various physical therapeutic modalities, such as massage, stretching, and heat, prove effective in alleviating cervical myofascial pain and reducing sensitivity in trigger points and improvement in function.	***
Rai, Ranjan, Misra, Panjwani (2016) [30].	Randomized Controlled Trial (RCT)	Therapeutic ultrasound and transcutaneous electrical nerve stimulation	Therapeutic ultrasound and transcutaneous electrical nerve stimulation effective for myofascial pain management.	**
Melis, Di Giosia , Zawawi (2022) [31].	Systematic Review	Oral myofunctional therapy	Oral myofunctional therapy beneficial for treating temporomandibular disorders (TMD).	***
de Felício, Melchior, da Silva (2010) [32].	Randomized Controlled Trial (RCT)	Orofacial myofunctional therapy	Orofacial myofunctional therapy shows positive effects on temporomandibular disorders compared to control group	***
Truelove, Huggins, Mancl, Dworkin (2006) [33].	Randomized Controlled Trial (RCT)	Traditional, low-cost, and splint therapies	Traditional, low-cost, and splint therapies effective in randomized controlled trial for temporomandibular disorder.	***
Wassell, Adams, Kelly (2006) [34].	Randomized Controlled Trial (RCT)	Stabilizing splints in general dental practice	Stabilizing splints show positive outcomes in treating temporomandibular disorders over a one-year follow-up.	***
Scopel, Alves da Costa, Urias (2005) [35].	Observational study	Electromyographic study of Masseter and anterior temporalis muscles in extra-articular myogenous TMJ pain patients compared to asymptomatic and normal population	Electromyographic study reveals differences in masseter and anterior temporalis muscles in TMJ pain patients compared to asymptomatic individuals.	***
Türp, Komine, Hugger (2004) [36].	Systematic Review	Efficacy of stabilization splints for masticatory muscle pain	Stabilization splints efficacy examined through qualitative systematic review for managing masticatory muscle pain.	***
Kreiner, Betancor, Clark (2001) [37].	Review	Occlusal stabilization appliances	Evidence supports efficacy of occlusal stabilization appliances in the management of temporomandibular disorders.	***
Alencar Jr, Becker (2009) [39].	Double-blind controlled clinical trial	Evaluation of different occlusal splints and counseling in the management of myofascial pain dysfunction	The findings indicated that all three types of appliances (hard, soft, or non-occluding occlusal splints) paired with counseling demonstrated equal effectiveness in reducing both the Modified Symptom Severity Index (Mod-SSI) and tenderness to palpation.	***
Munguia, Jang, Salem, Clark, Enciso (2018) [40].	Systematic Review and Meta-analysis	Low-level laser therapy (LLLT)	The results of this systematic review indicate that Low-level laser therapy (LLLT) appears to be efficacious in alleviating pain among individuals with myofascial pain associated with temporomandibular disorders, supported by evidence of moderate quality.	.***

Limitations

It is important to recognize the limitations when interpreting the findings of this review. These limitations included the lack of a rigorous and systematic approach to study selection, data extraction, and analysis can lead to a less objective synthesis of evidence. A notable limitation in the existing literature is the lack of comprehensive comparisons regarding treatment duration between these modalities. Moreover, physical therapeutic modalities like TENS, ultrasound, spray, and stretch have also shown marked improvement in pain, but further research is required to establish evidence-based treatment.

## Conclusions

MFP is due to TrPs in the muscles. Many treatment modalities are effective for the management of MFP. Mostly, patients are treated with analgesics and muscle relaxants that reduce the symptoms of MFP. Dry needling and TrP injections are the interventional treatment options; some studies also support such types of treatment. Our findings support the use of a combined approach that integrates both pharmacological and non-pharmacological strategies for the holistic management of TrPs. For acute cases, the effectiveness of analgesics, muscle relaxants, dry needling, and TENS has been demonstrated. Future robust research including a systematic review and metanalysis is required to establish an evidence-based management of MFP.
